# Actigraphy in Post Traumatic Stress Disorder 

**Published:** 2014

**Authors:** Imran S. Khawaja, Ali Madeeh Hashmi, Muhammad Awais Aftab, Joseph Westermeyer, Thomas D. Hurwitz

**Affiliations:** 1 Imran S. Khawaja, MD, FAASM,Medical Director, Minnesota Regional Sleep Disorders Center at Hennepin County Medical Center, Assistant Professor of Psychiatry, University of Minnesota School of Medicine.; 2 Ali Madeeh Hashmi, MD, Associate Professor of Psychiatry, King Edward Medical University/Mayo Hospital, Lahore, Pakistan.; 3 Muhammad Awais Aftab, MBBS, Resident in Psychiatry, Hamad Medical Corporation, Qatar.; 4 Joseph Westermeyer, MD, PhD, Minneapolis VA Medical Center, University of Minnesota, Minneapolis, United States.; 5 Thomas D. Hurwitz, MD, Minneapolis VA Medical Center, University of Minnesota, Minneapolis, United States.

**Keywords:** Actigraphy, PTSD, Sleep, Insomnia, Review Article

## Abstract

Patients with posttraumatic stress disorder (PTSD) frequently complain of sleep disturbances such as insomnia and nightmares. Evaluation of sleep disturbances is often difficult due to the subjective nature of the complaints. Polysomnography (PSG) and other sleep studies are generally not indicated in the evaluation of insomnia or nightmares associated with PTSD. Actigraphy, (electronic activity monitoring) has been used in research to evaluate sleep disturbances in patients with PTSD. We reviewed the literature on the use of actigraphy in evaluation of sleep problems in patients with PTSD.

***Methods: ***A literature search for articles on the topic was conducted on PubMed using the search algorithm (actigraphy[Title/Abstract] OR actigraphic[Title/Abstract]) AND PTSD[Title/Abstract]. Out of 11 search results, 9 studies in which application of actigraphy had relevance to the primary objective and outcome in PTSD patients with sleep problems were selected for review. We also handpicked one additional article from personal communication with our colleagues who have done some of these studies.

***Conclusion: ***Actigraphy has been used to evaluate circadian rhythm sleep disorders. Use of actigraphy in psychiatry clinics is uncommon. There is no data to support that there are specific actigraphic sleep related findings in PTSD patients. However, it can be a useful tool to complement the use of sleep diaries when assessing sleep and wake patterns in patients with PTSD.

## INTRODUCTION

Insomnia and nightmares are common complaints of patients with post traumatic stress disorder (PTSD) in addition to wakeful symptoms of re-experiencing traumatic events and avoidance of stimuli associated with the trauma. Sleep disturbance is a frequent, though often neglected part of the therapy in PTSD.^1^ There is increasing evidence of emergence of insomnia following trauma as a predictor of subsequent development of PTSD.^2^

Evaluation of insomnia is often challenging because of the subjective nature of the complaint. Patients can mistakenly misperceive sleep or wakefulness due to dissociation and sleep fragmentation.^3^ Collateral information is most valuable in addition to clinical history when evaluating sleep complaints. However, many individuals with PTSD do not have bed partners or roommates. Sleep logs are useful for evaluation of insomnia, but they do not give objective data. Because Polysomnography (PSG) is generally not useful nor indicated for evaluation of insomnia in PTSD, a simpler objective measure, actigraphy has been used to research settings to get subjective data about sleep.

Polysomnographic studies in PTSD patients have shown unclear findings.^4^ Based on meta-analysis, Kobayahi at al report an association between PTSD and increased Rapid Eye Movement (REM) density (a measure of rapid eye movement frequency during REM sleep), decreased stage N3 (“deep”) sleep, and increased stage N1 (light) sleep as compared with healthy subjects.^4^ These non-specific findings are not uniformly shown in all studies. Consequently routine PSG is not indicated in the clinical assessment of PTSD unless other sleep disorders such as obstructive sleep apnea or parasomnias are suspected. Many patients with PTSD sleep more comfortably in a sleep laboratory as it is perceived as safe and “protected” by the staff.^5^ In addition sleep complaints as perceived by patients with PTSD may also vary over periods of time, so that one night of data collection may be inadequate. This suggests that in-home monitoring, such as actigraphy may be helpful in documenting objective sleep disturbance in PTSD patients.

***Description of Actigraphy:***

Actigraphy, a technique of recording and measuring body or limb movements, can be used to evaluate sleep in combination with sleep diaries.^6^ Activity monitors (actigraphs) are small-computerized gadgets that record and store data caused by movements. Actigraphs can record continuously for up to several weeks. Different scoring algorithms can generate reliable estimations of sleep and wake periods. The device is typically worn on the non-dominant wrist for a couple of weeks. A computer download produces a pictorial and quantifiable report of movements per unit time that can be designated as sleep or wakefulness with good reliability.

Actigraphy has been validated and is reliable for assessment of sleeptime, but reliability for quantification of sleep onset latency and daytime sleepiness is low^6^. Actigraphy is most useful for description of sleep wake cycles, which can be useful in assessment of circadian rhythm disorders and treatment outcomes. It is important to ask individuals to collect subjective data each day in a sleep-wake diary to complement the objective activity data collected from the actigraphs. This helps with determination of time of going to bed, awakenings during the night and in the morning, and determines the limits of actual time in bed. This information can be used to determine the intervals used to define “time in bed” that are then scored as sleep or wake by movement count data. The diary can also be used as a backup if the device stops working and may help distinguish relatively motionless wakeful time from sleep. Other important relevant information may also be collected, such as medication administration, exercise or daytime naps.

There are several advantages of actigraphy, which include the following: Its coverage of extended periods of time, cost effectiveness, and ease of use in the natural environment. Its main disadvantage is that it cannot describe cerebral sleep-wake state as well as an EEG.

Though actigraphy has been used widely in research settings its clinical utility has been established gradually over time. According to the practice parameters established by the American Academy of Sleep Medicine^7^ actigraphy is indicated as a method to characterize circadian rhythm patterns or sleep disturbances in individuals with insomnia, including insomnia associated with depression. It was also found to be useful for evaluating the response to treatment for patients with insomnia. Presently there are no specific recommendations regarding the use of actigraphy in PTSD patients with sleep difficulties.

This paper reviews existing literature on the use of actigraphy in assessment of sleep difficulties in patients of PTSD, with the aim of demonstrating how actigraphy could contribute to our understanding of sleep problems in PTSD patients.

## METHODS

A literature search for articles on the topic was conducted on PubMed using the search algorithm *(actigraphy [Title/Abstract] OR actigraphic [Title/Abstract]) AND PTSD [Title/Abstract]*. A total of 11 studies came up in the results. The abstracts of these papers were examined. Studies in which application of actigraphy had relevance to the primary objective and outcome in PTSD patients with sleep problems were included. Studies in which actigraphy was used as an assessment tool but had no relevance to the primary objective and outcome of the study were excluded. No time constraints with regards to the publication of research were applied. Based on these inclusion and exclusion criteria, 9 studies out of the 11 were selected for review. In addition, we also considered some relevant studies not indexed in PubMed which were pointed out to us by our colleagues who have done work in this area, and from these one additional study was selected for review.

***Review of Literature:***

Among the 10 studies selected for this review, 4 studies examined objective (actigraphic) evidence of sleep disturbance in PTSD patients with sleep complaints; 2 studies^3^^,^^8^ found no actigraphic evidence while 2 studies^9^ did. One study^10^ investigated subjectively and objectively measured sleep with and without posttraumatic stress disorder and trauma exposure, and found that discrepancies between subjectively and objectively measured sleep parameters are not associated with trauma exposure or PTSD. Another study^11^ examined the reliability of sleep log data versus actigraphy in PTSD patients, and found it to be unreliable, especially with regards to the number of awakenings. Yet another study^12^ measured the correlation of actigraphic sleep disturbance with daytime sleepiness in PTSD patients, and found no correlation. One study^13^ examined autonomic activation during sleep in PTSD using an innovative technique of mattress actigraphy. One study^14^ used actigraphy to compare PTSD patients with and without mild traumatic brain injury. Another study^15^ evaluated the response to long-term Zopiclone in improving sleep quality in PTSD patients. One study^16^ found that the number of awakenings was higher in younger aged patients with PTSD as compared to the older ones.

***1) Actigraphic evidence of sleep disturbance in PTSD patients:***

Dagan et al.^3^ have examined actigraphic findings during sleep at home in 16 men with DSM III-R defined PTSD and 11 male non-PTSD controls. The PTSD group included randomly chosen Lebanon war veterans. The control group included veterans of that war without PTSD. Interestingly, PTSD patients did not have poorer actigraphic sleep time than controls, despite reporting poorer subjective sleep. The authors proposed that PTSD patients might fail to correctly assess their sleep. The concept of “sleep state misperception” or “paradoxical insomnia” in the newer diagnosis in the International Classification of Sleep Disorders (ICSD II) represents a similar phenomenon of discrepancy between subjective and objective findings of insomnia.

The idea that the problem in PTSD is distorted sleep perception rather than sleep disturbance is supported by another actigraphic study by Klein et al.^8^ in which 102 motor vehicle collision (MVC) survivors were followed for one year after the time of the incident. Nineteen subjects hospitalized for elective surgery were in the comparison group. Diagnosis of PTSD in the cohort was made using structured clinical interview (SCID) at 12 months after the collision. A 48-hour actigraphic recording was obtained at one week, three months and 12 months. While MVC survivors with PTSD reported markedly poorer sleep as reflected by significantly higher scores on the mini-Sleep Questionnaire, there were no significant differences between the three groups (MVC survivors with PTSD, MVC survivors without PTSD, Comparison group) on the actigraphic measures that were largely normal.

Contrary to the results of the above 2 studies, Calhoun et al.^9^ examined sleep disturbance in women with PTSD in their home environment. Results from actigraphy on 30 cases and 22 controls with three nights of monitoring indicated that women with PTSD had poorer sleep efficiency, increased sleep latency, and more restless sleep. Actigraphy measures were moderately correlated with self-report sleep-log data (but were unrelated to scores on the Pittsburgh Sleep Quality Index (PSQI), providing evidence that women with PTSD have objectively measured sleep disturbance in their normal home environment.

A 2012 study by Kobayashi et al.^10^ challenged the notion that individuals with PTSD over report their sleep disturbances, not by showing that the sleep disturbances are objectively valid, but rather those discrepancies between subjectively and objectively measured sleep parameters are not associated with PTSD. They used PSG recordings in a sleep laboratory and actigraphic recordings in participants' homes to study one hundred three African Americans with and without trauma exposure and PTSD. Participants, irrespective of their trauma exposure or PTSD status, underestimated wake after sleep onset (WASO) in the sleep diary and questionnaire relative to actigraphy and overestimated sleep onset latency (SOL) in the diary relative to PSG. Total sleep time (TST) diary estimates did not differ from the actigraphy measure among participants with current PTSD, in contrast to those without current PTSD who overestimated TST.

A recent study by Khawaja et al.^16^ showed that in patients with lifetime PTSD, younger age was associated with increased awakenings, possibly secondary to increased sleep difficulties early in the course of PTSD and gradual reduction in PTSD symptoms over time.

***2) Reliability of sleep log data versus actigraphy in PTSD:***

Westermeyer et al^11^ compared actigraphic and subjective sleep diary data for 241 nights among 21 veterans with lifetime PTSD. Actigraphic sleep minutes per night were, on average, 51 minutes longer than self-reported sleep minutes on sleep diaries. Total intra-class correlation between actigraphy and sleep logs was 0.588. In the same study, correlation between self-reported awakenings on the sleep logs and apparent actigraphic awakenings were calculated after excluding nights when there was failure to indicate sleep duration in the sleep diaries. Actigraphy showed 3.6 times more awakening episodes than did sleep diary information. This odds ratio differed greatly from one person to another, ranging from 2 to 12 times, leading the authors to suggest that self-reported awakenings are not reliable for scientific studies of individuals with PTSD. It is conceivable that patients moved sufficiently in their sleep to make movements that could appear as if an "awakening response" in the actigraphy. This study was limited by the inclusion of veterans receiving care for lifetime as opposed to current PTSD with sleep symptoms.

***3) Correlation of Actigraphy Measures with Daytime Sleepiness in PTSD:***

In another article, Westermeyer et al.^12^ looked at the same cohort to assess the correlates of daytime sleepiness in patients with lifetime PTSD, with Epworth Sleepiness Scale as the primary outcome measure. Neither sleep duration nor awakenings per night, as measured by actigraphy and sleep logs, were associated with the mean Epworth Sleepiness Scale score in this population.

***4) Autonomic activation during sleep in PTSD:***

Woodward and colleagues^13^ used a novel approach to examine movement in bed during sleep. Large and small movements of the sleeping individual activated accelerometers embedded in the mattress topper. With supplemented kinetocardiogram technology, they were able to assess movement and autonomic variability (autonomic activity is not recorded during a normal actigraphy). They reported on 59 non-veterans who were divided into the following groups: With PTSD, PTSD and panic disorder, panic disorder alone, and normal controls. Screening for other sleep disorders was done by PSG. In patients with PTSD with and without co morbid panic disorder, heart rate during sleep was higher than in control subjects (p<.05). Heart rates of those with panic disorder alone did not differ from controls. PTSD patients exhibited significantly longer periods of actigraphic sleep time and time in bed as compared to either panic disorder group or PTSD and panic disorder group. The PTSD-only subjects showed contradictory indicators, i.e. tonically elevated sympathetic tone with extended sleep periods.

Overall the study showed that non-veteran patients meeting criteria for PTSD alone had increased autonomic activation (not relevant to general actigraphy) co-occurring with prolonged actigraphic sleep periods, a finding which seems counterintuitive.

***5) PTSD with mild traumatic brain injury:***

Wallace et al.^14^ compared sleep characteristics of three groups of US Army veterans from Afghanistan and Iraq: healthy sleepers, those with insomnia associated with PTSD and mild traumatic brain injury (mTBI), and those with insomnia associated with PTSD alone. Participants undertook polysomnography (PSG) with 2 weeks of actigraphy and sleep diaries. Subjective daytime sleepiness was reported to be significantly greater in PTSD subjects with mTBI compared to the other two groups, while having a significantly shorter wake after sleep onset.

***6) Assessing treatment response:***

Alderman and Gilbert^15^ studied the effects of the long-term use of zopiclone, in patients with combat-related PTSD with a 6 month follow-up study with 26 participants. Actigraphic measurements were consistent at baseline and follow-up, with similar mean sleep efficiency scores at baseline and after 6 months of treatment with zopiclone (69.6 +/- 12.7% at baseline; 71.33 +/- 19.0% at follow-up), indicating a low efficacy. This study exemplifies the potential use of actigraphy in evaluating response to treatment for PTSD patients with sleep problems.

***7) Relevance to Pakistan:***

Pakistan has been an active participant in the so-called ‘War on Terror’ since about 2001. This conflict has resulted in close to 50,000 casualties (combined civilian and military).^17^ The Pakistan Army by some estimates, has lost over three thousand troops, equivalent to two full brigades in this conflict.^18^ This count does not include soldiers and civilians who have suffered significant injuries or other incidents^19^ which would predispose them to PTSD and its attendant complications. In spite of this, there are no published studies on the incidence, prevalence or risk factors of PTSD in Pakistani soldiers or civilians, and associated sleep disturbances. This study underlines the urgent need for such studies to be conducted, and the role actigraphy can play in them.

## CONCLUSION

Actigraphy can help with the evaluation of circadian rhythm sleep disorders, like delayed sleep phase syndrome, advanced sleep phase syndrome and shift work. The use of this device to assess sleep problems in clinical psychiatric setting is still uncommon. However, in patients with PTSD and other psychiatric disorders, it can be used to characterize circadian rhythm and sleep patterns. It is particularly useful in patients who are not compliant with sleep diaries. There is no data to support any specific actigraphic findings related to PTSD and is not indicated other than characterizing their sleep pattern and helping them understand their own sleep pattern. 

## Author’s contribution:

**ISK **was involved in conception of the study, literature review, manuscript writing, and final approval. **AMH and MAA **contributed to methodology, literature review, analysis, manuscript writing and editing. **JW and TH **were involved in analysis of selected papers for review, along with substantial contributions to drafting the manuscript.
